# Landscape Simplification Constrains Adult Size in a Native Ground-Nesting Bee

**DOI:** 10.1371/journal.pone.0150946

**Published:** 2016-03-04

**Authors:** Miles Renauld, Alena Hutchinson, Gregory Loeb, Katja Poveda, Heather Connelly

**Affiliations:** 1 Department of Entomology, Cornell University, Ithaca, New York, United States of America; 2 Department of Entomology, New York State Agricultural Experiment Station, Cornell University, Geneva, New York, United States of America; Ghent University, BELGIUM

## Abstract

Bees provide critical pollination services to 87% of angiosperm plants; however, the reliability of these services may become threatened as bee populations decline. Agricultural intensification, resulting in the simplification of environments at the landscape scale, greatly changes the quality and quantity of resources available for female bees to provision their offspring. These changes may alter or constrain the tradeoffs in maternal investment allocation between offspring size, number and sex required to maximize fitness. Here we investigate the relationship between landscape scale agricultural intensification and the size and number of individuals within a wild ground nesting bee species, *Andrena nasonii*. We show that agricultural intensification at the landscape scale was associated with a reduction in the average size of field collected *A*. *nasonii* adults in highly agricultural landscapes but not with the number of individuals collected. Small females carried significantly smaller (40%) pollen loads than large females, which is likely to have consequences for subsequent offspring production and fitness. Thus, landscape simplification is likely to constrain allocation of resources to offspring through a reduction in the overall quantity, quality and distribution of resources.

## Introduction

Pollinators, in particular bees, are critical ecosystem service providers, responsible for the pollination of 87% of angiosperm plants [1] including more than 70% of crop species [2]. Although declines in bee populations have been documented [3,4], the causes of these declines are poorly understood and likely multifaceted [5].

Agricultural intensification resulting in the simplification of environments at the landscape scale greatly alters the resources available for bees and has been demonstrated to alter the abundance and distribution of a number of taxa [6,7,8]. Landscape simplification is associated with a transition of perennial natural habitats to arable fields, destruction of edge habitats and simplification of overall land-use types resulting in a reduction in habitat connectivity and greater fragmentation and isolation of remaining natural habitat patches [9].

As central place foragers, landscape composition is likely to have a strong influence on a female bee’s ability to locate and obtain quality nesting and provisioning resources. Agricultural intensification resulting in the reduction and fragmentation of the natural and semi-natural habitat patches that bees rely on for floral and nesting resources may increase foraging times [10,11], decrease the quality of resources [12] and increase pesticide exposure [13]. As floral and nesting resources become scarce, bees are more likely to use resources farther from the nesting site [14]. Longer foraging distances over a fixed nesting period equate to fewer overall foraging trips and therefore lower resource acquisition [15]. Potentially, longer foraging trips may also lead to a greater risk of adult predation as well as increasing the nest’s vulnerability to cleptoparasitism [16,17, 18] and adult exposure to pesticides. The likelihood of females collecting pesticide-laden pollen to provision brood cells may also be higher in simplified landscapes where pesticide use increases ([13], but see [19]).

Parental investment in offspring is optimized to maximize fitness through tradeoffs between the size, number and sex ratios of offspring [20, 21, 22]. Ecological and environmental conditions such as resource distribution [14, 14], abundance [23, 24], quality [25] and competition [16] can alter or constrain the tradeoffs in parental offspring investment. The cost of acquiring resources varies in changing environments and influences how parents allocate resources to offspring [26, 27]. Roulston and Cane [25] reported that offspring of the ground nesting bee *Lasioglossum zephyrum* grew larger on a high protein pollen diet, and although females responded to changes in floral resource abundance by altering provision mass size they did not compensate in response to lower pollen quality, suggesting that lower quality resources in agriculturally intensified landscapes could play a role in reducing body size.

Hymenopterans in particular have been a fertile ground for developing and testing theories on maternal offspring investment, as they tend to be strongly sexually dimorphic in size and females have control through haplodiploidy over the primary sex ratio of offspring. Bees provide an excellent system to study maternal resource allocation tradeoffs since all food consumed by the offspring prior to adulthood is provided by the mother and the amount of food provisioned to individual offspring is correlated to the subsequent size of the progeny at maturity [28, 29, 30, 23, 31]. Additionally, the heritability of body size appears low [32] and may be adaptively adjusted by females in response to seasonally changing resource levels [27].

When resources vary in space and time, females should alter the optimal amount of resources provisioned per offspring in order to maximize fitness [26, 27]. Seasonal changes in weather conditions and in resource availability have been shown to influence adult bee size in the following year in both solitary [33, 34] and social bee species [35]. Previous studies have relied on managed bee populations to isolate individual factors affecting maternal resource allocation such as foraging distance [15] and specific resource levels [23, 24] but in reality these factors are likely to be correlated and also linked to additional factors such as resource quality, competition and parasite pressure. Measurements of bee size and number in natural populations allows for the assessment of the cumulative effects of these factors simultaneously.

Here we focus on the relationship between landscape scale agricultural intensification and the size and number of individuals of the wild ground nesting bee species, *Andrena nasonii*. We hypothesize that changes in resource quantity and quality associated with gradients in agricultural land-use intensity constrain maternal investment tradeoffs such that 1) smaller individuals and 2) fewer individuals are produced in highly agricultural landscapes. Further, we explore the ecological consequences of a smaller bee size and test the prediction that smaller female bees carry smaller pollen loads than larger females.

## Methods

### Study System

Though bees in the family Andrenidae are among the most common and speciose of the vernal community, detailed life history information is sparse for the majority of species. *A*. *nasonii* Robertson 1895, is a polylectic bee with a short flight period in early spring [36]. It is an abundant floral visitor to many early spring fruit crops including apple [37], blueberry [38] and strawberry [39]. Although little is known about *A*. *nasonii* nesting biology specifically, bees in the genus *Andrena* are known to excavate nesting tunnels in the ground either singly or in aggregations but always with each female constructing and occupying a single nest. Nests are comprised of a simple tunnel up to 1 m in length ending in a small number of brood cells, which are sequentially provisioned with a mass of pollen mixed with nectar [40]. Following completion of the provision, a single egg is laid on the mass and the entrance to the brood cell is filled with soil [40].

### Landscape Parameters

We identified 16 farms in the Finger Lakes Region of New York USA along a gradient in landscape complexity and established standardized 100m^2^ strawberry (*Fragaria x ananassa*) plots of the variety “Jewel”. Plots were located on Cornell University research farms or on private farms (landowners gave permission to conduct the study at these sites) and varied in the cover of agricultural land use in the surrounding landscape. Radii of 750 m and 1 km were selected as strawberry pollinator populations, which are dominated by *A*. *nasonii*, have been shown to respond to landscape gradients at these scales [39]. Agricultural lands were defined as pastoral and cultivated crops and estimated from the 2013 & 2014 NASS Cropland Data Layers [41]. Natural and semi-natural habitats included forests, wetlands, scrublands, fallows, open and low intensity developed land (roadsides etc). Because many early season flowering crops provide key floral resources for *A*. *nasonii* and *A*. *nasonii* may use these habitats as well as pastures and grasslands as nesting habitats, we included in our analysis the cover of early season blooming crops as well as pastures and cultivated crops separately.

### Bee Size and Number

Bee specimens were collected using sweep nets along 50m transects once a week over four weeks in May of 2014 and 2015. Individuals were quickly killed in ethyl acetate killing jars and frozen (-20°C) until further processed. Species identification of each bee was verified using reference materials and DiscoverLife.org keys. Only five male *A*. *nasonii* were collected but were excluded from analyses given the small sample size. Two morphological measures of size were taken for each female bee: inter-tegular distance (ITD) and head capsule width using an ocular micrometer. These measures provide accurate estimates of bee size and have been used in a number of other studies [42, 43]

### Pollen Load

To estimate the effect of bee size on its ability to collect pollen resources, we randomly selected 12 individuals from the upper and 12 individuals from the lower quartiles of bees collected based on inter-tegular distances. These 24 specimens were placed into pre-weighed 2 ml eppendorf tubes with 1 ml of 65°C 70% ethanol and vortexed for 15 minutes. The bees were then washed with 0.5 ml of 25°C 70% ethanol to remove any remaining pollen from the body and the tubes were centrifuged for 5 minutes at 13,000 RPM. The ethanol was decanted from each tube and the tubes were placed on a 65°C heating block to dry until they had reached a stable weight (approx. 3 hours). The final weight of the tube was recorded to the nearest 0.0001g from which the initial weight of the tube was subtracted to estimate the total weight of the pollen.

### Statistical Analyses

Separate linear mixed effects models were fit using the nlme package [44] in R (v 3.1.0) [45] to determine the effects of landscape simplification on our measures of bee size (ITD and head capsule width) with each land cover variable as a fixed effect and sampling date within farm within year as hierarchical random effects. Land cover variables were correlated among themselves (S1 Table); thus, separate models were fit for each cover type and scale, and comparisons of model fit were made using AICc values. Models including with all agricultural lands at 750m (ITD AICc = 13.72) and 1km (ITD AICc = 11.83) were equivalent based on AICc. Therefore, the data for all other landscape variables are presented at the 1km scale. The effect of landscape simplification on abundance was tested with a linear mixed effects model with total number of individuals collected per site per year as the response variable and the most predictive land cover variable from the previous analysis as a fixed effect. Farm within year was included in the model as a hierarchical random effect. A t-test was used to determine whether pollen loads were different between the two bee size categories.

## Results

A total of 112 female *A*. *nasonii* were collected over the course of strawberry bloom in May 2014 and 2015. An average of 5 individuals were collected per site with a minimum of 1 and a maximum of 10. The inter-tegular distance of female *A*. *nasonii* ranged from 1.4615 to 1.9615 mm and the head capsule width ranged from 2.1538 to 2.7692 mm.

### Bee Size and Number

**Fig 1:**
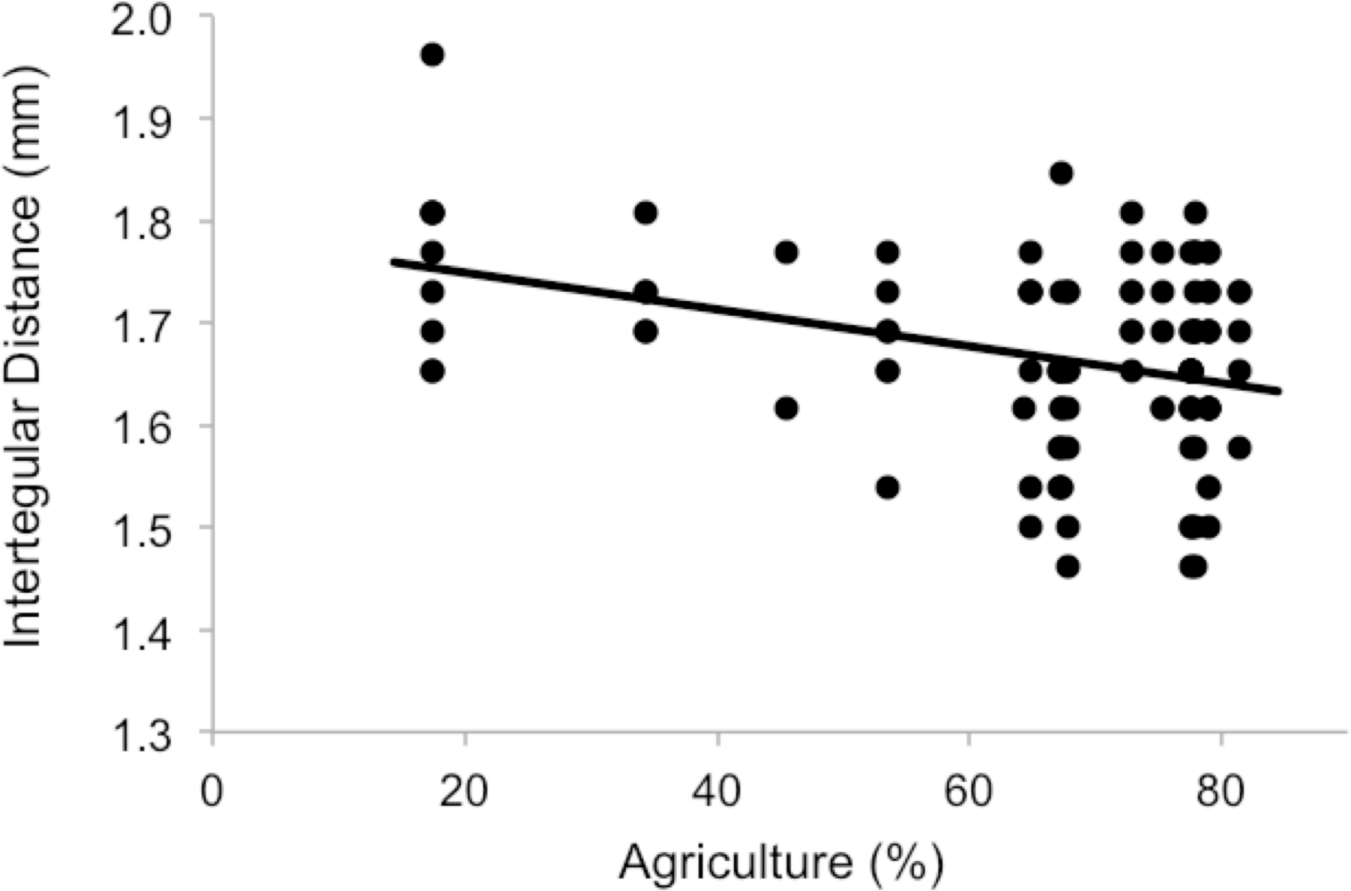
Inter-tegular distances of adult female *A*. *nasonii* in relation to the percentage of agricultural land uses within a 1 km radius from the collection sites.

**Fig 2:**
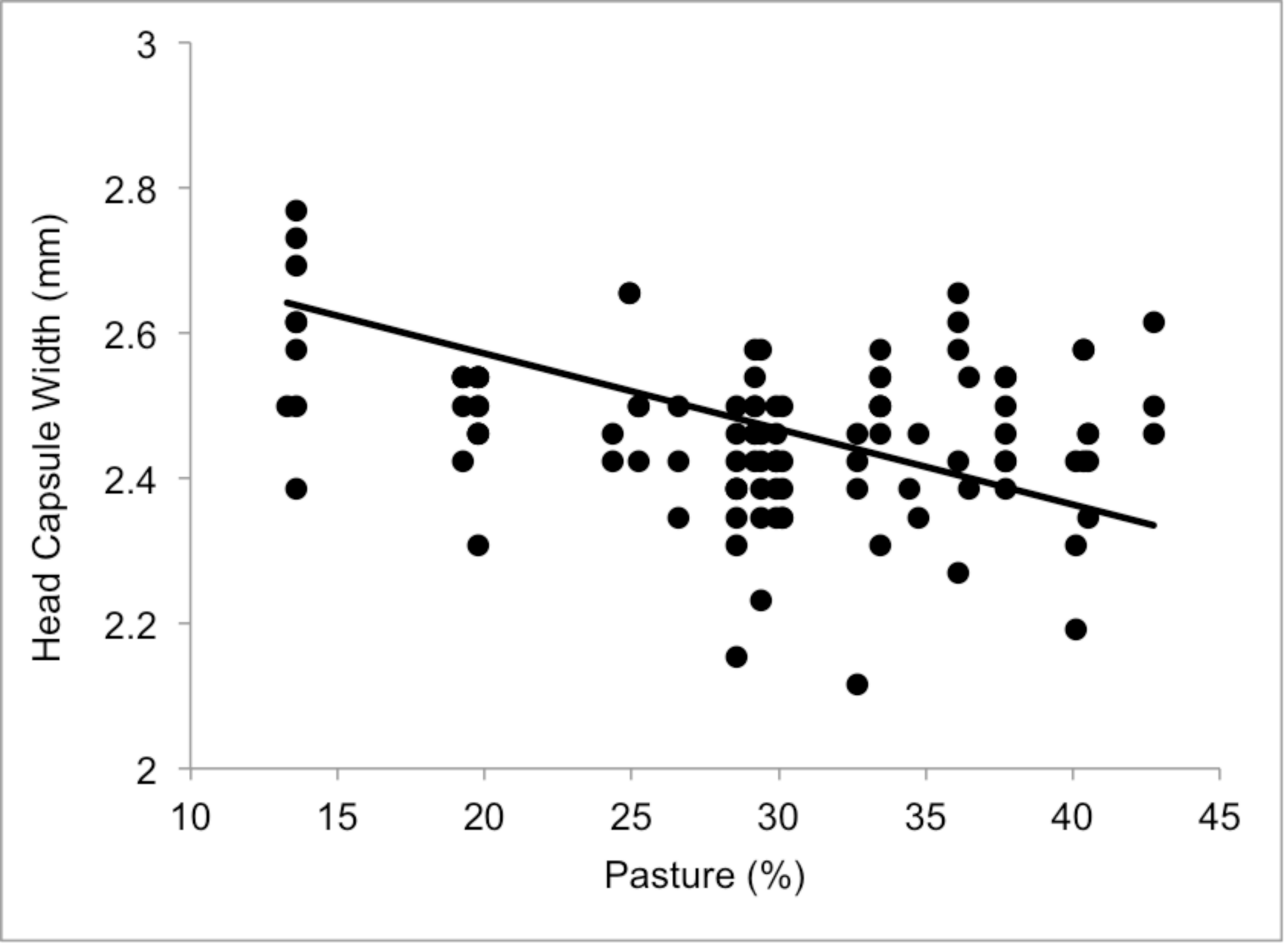
Head capsule widths of adult female *A*. *nasonii* in relation to the percentage of pasture land within a 1 km radius from the collection sites.

A significant positive correlation was found between inter-tegular distance and head capsule width (Pearson’s r = 0.67, p<0.001, N = 109). The top models describing the effects on ITD included all agricultural land use types at the 1 km scale and at the 750 m scale (Table 1). Increasing cover of agriculture at both scales had a negative effect on bee size, measured by ITD (1 km: *F*_(1,21)_ = 11.99 P = 0.002, Fig 1, 750 m: *F*_(1,21)_ = 9.94 P = 0.004). For every 10% increase in the percent agriculture at 1 km, ITD decreases by 0.047 mm. Although a similar effect of agriculture was found on head capsule width (*F*_(1,21)_ = 4.96 P = 0.036); the best model included only the percentage of pasture cover (*F*_(1,21)_ = 4.99 P = 0.036, Table 1, Fig 2).

**Table 1:** Model selection statistics for models describing inter-tegular distance and head capsule width as a function of percent agricultural land cover (cropland including pastures), cropland only, pastures only, natural and semi-natural cover (forest, fallows and open land), and percent cover of insect pollinated blooming crops at 750m and 1000m radii from the collection sites. The overall best model and competing models (AICc ≤ 2) are bolded. Asterisks (*) indicate models with significant (p <0.05) terms.

Response	AICc	ΔAICc	Scale	Metric	Coeff	Weight
Inter-tegular Distance	**11.83**	**0.00**	**1000m**	**All Agriculture**	-0.0047*	**0.464**
	**13.72**	**1.89**	**750m**	**All Agriculture**	-0.0040*	**0.181**
	14.28	2.45	1000m	Natural	0.0061*	0.136
	14.37	2.54	1000m	Cropland only	-0.0051*	0.180
	15.58	3.75	1000m	Pastures	-0.0074*	0.071
	18.00	6.17	1000m	Blooming Crops	-0.0039	0.010
Head Capsule Width	**26.09**	**0.00**	**1000m**	**Pastures**	-0.0091*	**0.505**
	28.40	2.31	1000m	All Agriculture	-0.0039*	0.159
	29.02	2.93	750m	All Agriculture	0.0034^(^*^)^	0.117
	29.59	3.50	1000m	Natural	-0.0041	0.088
	30.07	3.98	1000m	Cropland only	-0.0033	0.069
	30.30	4.21	1000m	Blooming Crops	-0.0030	0.062

We found no effect of either percent agriculture or pastures at the 1-km scale on the number of *A*. *nasonii* individuals collected per site within a given year (AG: *F*_(1,20)_ = 0.281 P = 0.60; PAST: *F*_(1,20)_ = 1.26 P = 0.27).

### Pollen load

**Fig 3:**
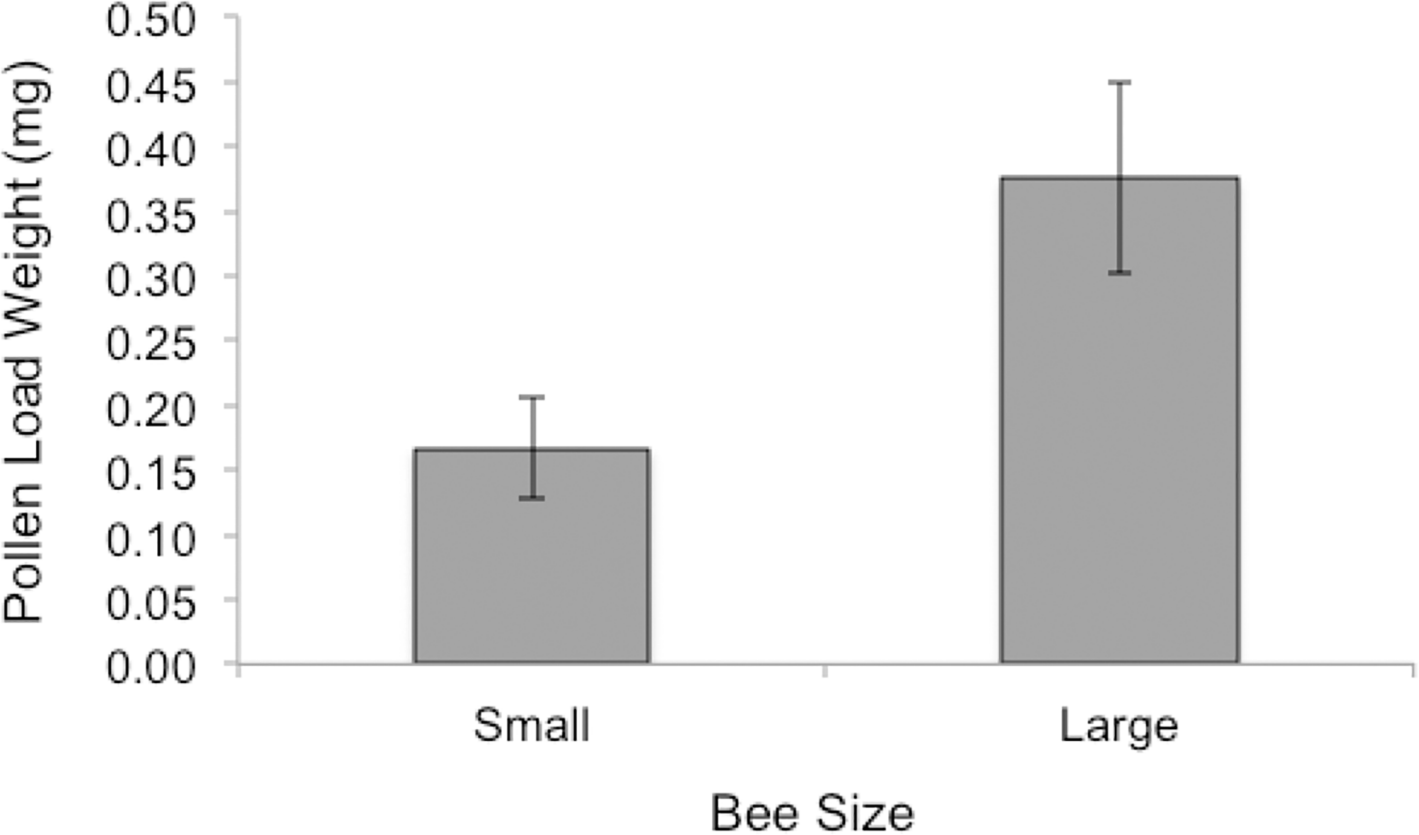
Average weight of pollen load (+/- SE) of small and large female *A*. *nasonii* collected while foraging on standardized strawberry plots.

Bee size was found to significantly impact the weight of pollen loads with large bees carrying heavier pollen loads than smaller bees (*t* = 2.4942, df = 17.193, p-value = 0.02309). Bees in the large category were 20% larger (mean = 1.788 +/- 0.0075 SE), on average, than bees in the small category (mean = 1.538 +/- 0.031 SE), and they carried 40% more pollen on average (Fig 3).

## Discussion

Understanding how current land use practices affect pollinator fitness is a crucial step towards proposing sustainable management practices that take into account pollinator health. Using a natural population of the wild bee *A*. *nasonii*, we show here that landscape scale variation in agricultural intensification was inversely related to the average size but not the number of field collected *A*. *nasonii* adults.

One explanation for the observed reduction in size could be an increased exposure to pesticide residues in highly agricultural landscapes. However, research in this area has been scarce. Morandin and Winston [46] reported no effect of exposure to multiple pesticides at field realistic doses on worker weight in two Bombus species. In this study and others however, insecticide exposure did result in a reduction in foraging efficiency. *Bombus terrestris* workers exposed to field realistic doses of imidicloprid collected 30% less pollen on average than control workers [12]. This reduction in foraging efficiency could lead to a reduction in the size of individuals in subsequent generations similar to the results observed in our study.

Resource quality is also likely to be impacted by landscape simplification, as agricultural intensification is associated with a change in plant communities and a shift toward less preferred plant species [14, 11, 47]. Both pollen nutrient content [25, 48] and chemical defenses [49, 50] are known to impact bee larval growth.

Landscape simplification may also limit the allocation of resources to offspring through a reduction in the overall quantity and distribution of available resources. The solitary bee, *Megachile rotundata* was found to reduce the size of female offspring and the total number of offspring produced when presented with experimentally reduced floral resources [24]. This effect may not simply be a consequence of reduced resources but may be an adaptive response to resource variability. Smaller offspring require fewer resources to produce, thus in a landscape with few floral resources small body size may lead to higher fitness. Indeed, multivoltine *Megachile apicalis* adjust female offspring body size in order to maximize performance under seasonally variable resource conditions [27].

Variation in the distribution of resources and habitat isolation associated with landscape simplification may increase foraging distances, which directly impacts the allocation of resources to offspring by reducing total quantity provisioned per unit time. *Osmia cornifrons* nesting in agricultural landscapes in the Central Valley of California preferred to collect pollen from native plant sources and were forced to forage further from nesting sites in areas isolated from natural habitats resulting in a decrease in offspring production and survival [14]. Increased foraging distance also indirectly increases the risk of predation [51] and nest parasitism [16, 17, 18]. Together these factors are likely to constrain the quantity or quality of provisions allocated by female *A*. *nasonii* and explain the observed reduction in size in simplified landscapes. While average bee size was reduced in highly agricultural landscapes, the total number of individuals collected remained constant, suggesting that females may be constrained in adjusting allocation of resources between offspring size and offspring number. It is possible however; that with decreasing cover of semi-natural habitat at the landscape scale, flowers in the local patch become more attractive to pollinators [52] thus leading to an overestimation of *A*. *nasonii* numbers in simplified landscapes. Recently, floral resource plantings incorporated in agricultural landscapes have been shown to increase the abundance of bees in adjacent habitats [53, 54]. The results of these studies highlight the importance of floral resources and suggest that increasing abundance and diversity of floral resources are likely to ease the constraints on maternal resource allocation to offspring; although, this prediction has not yet been explicitly tested.

In our study, large *A*. *nasonii* females carried significantly larger (40%) pollen loads than small females. These large females should therefore be capable of provisioning larger or a greater number of brood cells per unit time in landscapes with abundant resources [55, 56]. Because smaller bees require fewer resources to produce, small females may still provision equivalent number of smaller offspring in landscapes with limited floral resources. Large females should complete individual brood cells with fewer foraging trips and therefore should experience a reduced probability of nest parasitism [16, 17, 18] or desiccation of the incomplete provision mass, both of which are known to increase the overwintering mortality of brood [33]. However, producing smaller provision masses may also decrease time to completion and reduce risks associated with parasitism and desiccation. Similarly, though large females may be more likely to usurp the nests of smaller females [57, 55] when nest sites are limited, small females may have broader range of nest availability than larger females [27], especially in cavity nesting species.

The advantage of lower resource requirements may outweigh the costs associated with smaller body size in landscapes with limited resource availability. Although large females are likely to forage farther [58] and earlier in the day [59] than small females, large bodied bee species are declining at a greater rate that smaller species [60, 47], possibly due to greater pollen requirements [61]. Although the approach take in this study allows for the examination of multiple factors that vary across broad spatial scales, it is unclear whether the observed response of bee size to land use intensification represents a negative impact of reduced resources and/or increased pesticide exposure or an adaptive response. Future research efforts should focus on evaluating the performance of large and small *A*. *nasonii* females under varying resource conditions and on examining number of brood cells and quality of pollen masses in the nests of *A*. *nasonii* or similar wild bee species across a landscape gradient.

Although the number of individuals collected was similar across landscapes in our study, the reduction in average bee size may have cascading consequences for subsequent generations. At the population and community level, this trend may explain the pattern of reduced abundance and species richness of bees in highly agricultural landscapes [7, 60, 39]. These results contribute to our understanding of the causes of bee decline and provide insights into management strategies to improve pollinator health, such as increasing floral resources in simplified, highly agricultural landscapes.

## Supporting Information

S1 Table Matrix of Pearson’s Correlation values for landscape variables. Upper value is the correlation coefficient. Lower value is p-value.
(DOCX)
