# Supplementary material for: Landscape Simplification Constrains Adult Size in a Native Ground-Nesting Bee
Source: PLoS One. 2016 Mar 4;11(3):e0150946. doi: 10.1371/journal.pone.0150946 (PMC4778946; doi:10.1371/journal.pone.0150946)
Supplement: S1 Table — Upper value is the correlation coefficient. Lower value is p-value. (DOCX) [file pone.0150946.s001.docx]

S1 Table. Matrix of Pearson’s Correlation values for landscape variables. Upper value is the correlation coefficient. Lower value is p-value.

|  | **All Agriculture 750 m** | **All Agriculture 1 km** | **Cropland 1 km** | **Pastures 1 km** | **Blooming Crops 1 km** | **Natural 1 km** |
| --- | --- | --- | --- | --- | --- | --- |
| **All Agriculture 750 m** | X | 0.78 p<0.001 | 0.71 p<0.001 | 0.50 p<0.001 | 0.64 p<0.001 | -0.94 p<0.001 |
| **All Agriculture 1 km** | 0.78 p<0.001 | X | 0.68 p<0.001 | 0.48 p<0.001 | 0.60 p<0.001 | -0.96 p<0.001 |
| **Cropland 1 km** | 0.71 p<0.001 | 0.68 p<0.001 | X | 0.21 p=0.024 | 0.61 p<0.001 | -0.93 p<0.001 |
| **Pastures 1 km** | 0.50 p<0.001 | 0.48 p<0.001 | 0.21 p=0.024 | X | -0.09 p=0.33 | -0.32 p<0.001 |
| **Blooming Crops 1 km** | 0.64 p<0.001 | 0.60 p<0.001 | 0.61 p<0.001 | -0.09 p=0.33 | X | -0.76 p<0.001 |
| **Natural 1 km** | -0.94 p<0.001 | -0.96 p<0.001 | -0.93 p<0.001 | -0.32 p<0.001 | -0.76 p<0.001 | X |
